# Advance Care Planning Among Diverse U.S. Older Adults with Varied Cognition Levels

**DOI:** 10.1002/alz.084832

**Published:** 2025-01-09

**Authors:** Zahra Rahemi, Swann Arp Adams

**Affiliations:** ^1^ Clemson University, Greenville, SC USA; ^2^ University of South Carolina, Columbia, SC USA

## Abstract

**Background:**

Older adults from minority groups often experience elevated rates of chronic diseases and cognitive impairment, coupled with lower rates of engagement in advance care planning (ACP) and comfort care as they approach end of life. The complexities of ACP and end‐of‐life care become particularly pronounced in older adults with cognitive impairment. As cognitive and decisional capacity changes, healthcare decisions are commonly delegated to family members or proxies. This can present challenges, especially when life expectations are uncertain and discussions regarding ACP have not taken place. This study explores the disparities in ACP among diverse U.S. older adults with varying levels of cognitive function.

**Method:**

Secondary data analyses were performed utilizing the 2014 Health and Retirement Study. The outcome variables assessed were the possession of a living will, durable power of attorney for healthcare (DPOAH), and both a living will and DPOAH. Logistic regression models were applied to each outcome variable (living will, DPOAH, and both) with stratification based on cognition (dementia/impaired cognition versus normal cognition). Prediction variables encompassed race, ethnicity, rurality, marital status, gender, education, age, and social support.

**Result:**

Among the 17,698 respondents, 77.8% exhibited normal cognition, while 22.2% received a diagnosis of dementia or impaired cognition. Black and Hispanic participants, as well as younger individuals with lower levels of education, demonstrated a reduced likelihood of having a living will or both DPOAH and a living will. In the normal cognition group, race and ethnicity emerged as significant predictors of DPOAH, but this association was not observed in the dementia group. Additionally, rurality significantly predicted the presence of a living will and both DPOAH/living will for the normal cognition group, while such an association was not evident in the dementia/impaired cognition group.

**Conclusion:**

Our study revealed that individuals facing cognitive impairments exhibited lower rates of engagement in ACP. Notably, among the variables examined, race, ethnicity, rural residence, education, and age emerged as significant predictors of ACP in a national sample of older adults in the U.S. These findings underscore the importance of incorporating these sociodemographic factors into the design of interventional studies aimed at enhancing ACP and mitigating disparities.

